# Gallbladder torsion within incisional hernia: an original cholecystitis

**DOI:** 10.1186/s40064-015-1112-6

**Published:** 2015-07-01

**Authors:** Pierre Goubault, Kayvan Mohkam, Agnès Rode, Christian Ducerf, Jean-Yves Mabrut, Nicolas Golse

**Affiliations:** Digestive Surgery and Liver Transplantation Department, Croix-Rousse University Hospital, University Claude-Bernard Lyon-1, 103 Grande rue de la Croix Rousse, 69317 Lyon Cedex 04, France; Radiology Department, Croix-Rousse University Hospital, University Claude-Bernard Lyon-1, Lyon, France

**Keywords:** Gallbladder torsion, Incisional hernia, Cholecystectomy, CT-scan

## Abstract

Gallbladder torsion with ischemic wall necrosis is a rare condition, as gallbladder herniation. We describe here an original case of a patient with a symptomatic incisional hernia containing a gangrenous gallbladder twisted about its pedicle. We report preoperative findings on CT-scan and emergency surgical management.

## Background

Gallbladder torsion with ischemic wall necrosis is a rare condition, as gallbladder herniation. We describe here an original case of a patient with a symptomatic incisional hernia containing a gangrenous gallbladder twisted about its pedicle. This case represents diagnostic and therapeutic challenging.

## Case description

An 81-year-old woman was admitted in our institution for abdominal sepsis for 3 days. Her medical history included an obstructive left-sided colonic cancer 10 years ago, treated first by a discharge colostomy followed by open left-colectomy. On physical examination, there was a tender and painful mass in the right upper quadrant, corresponding to an incisional hernia at the site of the previous colostomy (inflammatory skin). The patient presented fever without occlusive symptom. Routine laboratory features showed an hyperleucocytosis (22.7 G/L) with an elevated c-reactive protein (343 mg/L) and an acute renal insufficiency. Hepatic tests were normal.

**Figure 1:**
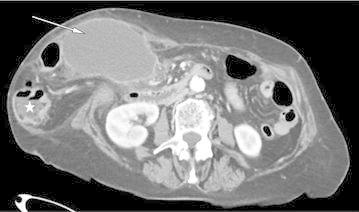
Pre-operative CT-scan. Incisional hernia containing the gallbladder (*arrow*) and digestive structure (*star*).

**Figure 2:**
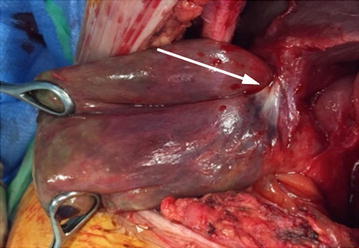
Intra-operative view. Note the twisted pedicle (*arrow*).

An emergency contrast-enhanced multislice helical-computed tomography (CT) scan revealed a large incisional hernia containing some digestive structures (satisfactory vascular enhancement of caecum and jejunum) and a thick-walled gallbladder (Figure 1). The diagnosis of ischemic cholecystitis in a non-strangulated incisional hernia was established. Emergency surgical exploration confirmed the incisional hernia, containing small bowel and colon without any strangulation. The gallbladder was gangrenous and twisted about its pedicle (180°) (Figure 2). After untwisting, an anterograde cholecystectomy with intraoperative cholangiography was performed. Due to the infectious risk, the fascial repair was performed without prosthesis implantation. Recovery was uneventful and the patient was discharged on postoperative day 9. At the 3 months follow-up, she was asymptomatic with no incisional hernia recurrence.

## Discussion

Gallbladder torsion (or volvulus) is a rare condition (400 cases reported in the literature), predominantly occurring in female (sex ratio 3:1) and the average age of incidence is 70 (Nakao et al. 1999). Elongation and relaxation of its mesentery leads to visceroptosis and favors twist about its pedicle. This is facilitated by the absence of fixation of the gallbladder fundus to the liver. Precipitating factors reported in the literature are varied such as important weight loss, sigmoid volvulus, constipation or diarrhea, cholelithiasis or gallstones (Marano et al. 2002). Clinical presentation may be different either the twist is under 180° (incomplete), with progressive abdominal pain, or over 180° (complete), with acute onset of abdominal pain, nausea and vomiting. Torsion of the gallbladder neck leads to mural ischemia consecutive to cystic duct obstruction and/or cystic artery strangulation (Lemonick et al. 2006; Alevizos et al. 2012).

**Figure 3:**
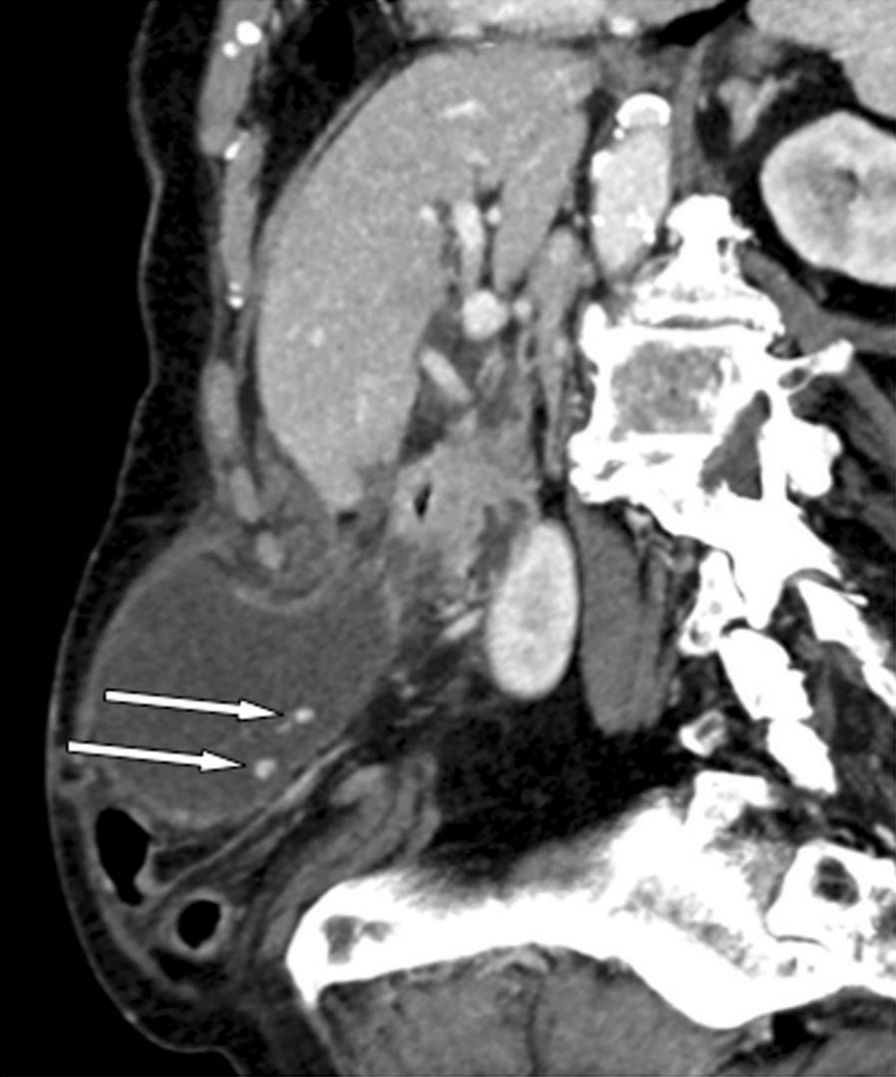
CT-scan pre-operative view. Note the absence of gallbladder fundus fixation, the presence of gallstones (*arrows*). On coronal planes we suspected a volvulus (*whirl-sign*).

Pre-operative diagnosis is made in only 10% of cases. A thickened wall is often noted on the CT scan, with poor enhancement on injected series. A “whirl-sign” can additionally be described (Figure 3). Gallstones could also be observed but they are not classically thought to be responsible for the symptoms. CT-scan coronal planes may be helpful to evoke diagnosis of gallbladder volvulus. On ultrasonography, a thickened wall is present with poor flow-signal on Doppler (Rosenblum et al. 2013; Pijpers et al. 2007).

The gallbladder can occasionally be found within an abdominal hernia, classically without volvulus associated. Only one case of gallbladder volvulus within an incisional hernia has been previously described, but it was a parastomal herniation (Rosenblum et al. 2013). Our observation is singular since it is the first case of gallbladder torsion within incisional hernia, without any mechanical complication of the hernia.

Gallbladder torsion requires an emergency surgical procedure (cholecystectomy) since percutaneous cholecystostomy is not adapted in this particular situation (no transhepatic passage). A direct surgical approach on the incisional hernia confirmed the diagnosis and the integrity of digestive structures. It allowed both the treatment of the gallbladder torsion and the repair of the fascial defect at the same time. Due to the high rate of postoperative infections in these septic conditions, fascial repair was performed without any synthetic mesh. A biologic mesh could have been discussed (Ventral Hernia Working Group et al. 2010). However, the lack of strong scientific evidence, the high cost and the poor availability of these prostheses raise issues as to their interest in emergency surgery.

## Conclusion

Albeit exceptional, gallbladder torsion should be considered in acute complicated cholecystitis with abnormal imaging findings (herniation, twisted pedicles or floating gallbladder). Ultrasonography and CT-scan may be helpful to perform the correct diagnosis before surgical management.

## Abbreviation

CT: computed tomography

## References

[CR1] Alevizos L, Stamou KM, Tsamis D, Pattas M, Menenakos E, Zografos GC (2012). Gallbladder volvulus as a cause of an acute abdomen in a 95-year-old patient. Am Surg.

[CR2] Lemonick DM, Garvin R, Semins H (2006). Torsion of the gallbladder: a rare cause of acute cholecystitis. J Emerg Med.

[CR3] Marano A, Yahchouchy-Chouillard E, Spinelli R, Ianelli A, Aura T, Fingerhut A (2002). Gallbladder torsion: report of four cases and review of the literature. Asian J Surg.

[CR4] Nakao A, Matsuda T, Funabiki S, Mori T, Koguchi K, Iwado T (1999). Gallbladder torsion: case report and review of 245 cases reported in the Japanese literature. J Hepatobiliary Pancreat Surg.

[CR5] Pijpers M, Herzebroek EJ, Coene PP, Beerman H, Vroegindeweij D (2007). Herniation of the gallbladder through the abdominal wall. Australas Radiol.

[CR6] Rosenblum JK, Dym RJ, Sas N, Rozenblit AM (2013). Gallbladder torsion resulting in gangrenous cholecystitis within a parastomal hernia: findings on unenhanced CT. J Radiol Case Rep.

[CR7] Breuing K, Butler CE, Ferzoco S, Franz M, Hultman CS, Ventral Hernia Working Group (2010). Incisional ventral hernias: review of the literature and recommendations regarding the grading and technique of repair. Surgery.

